# Validation of a machine learning algorithm for early severe sepsis prediction: a retrospective study predicting severe sepsis up to 48 h in advance using a diverse dataset from 461 US hospitals

**DOI:** 10.1186/s12911-020-01284-x

**Published:** 2020-10-27

**Authors:** Hoyt Burdick, Eduardo Pino, Denise Gabel-Comeau, Carol Gu, Jonathan Roberts, Sidney Le, Joseph Slote, Nicholas Saber, Emily Pellegrini, Abigail Green-Saxena, Jana Hoffman, Ritankar Das

**Affiliations:** 1 Cabell Huntington Hospital, Huntington, WV USA; 2 Marshall University School of Medicine, Huntington, WV USA; 3 Dascena, Inc., P.O. Box 156572, San Francisco, CA 94115 USA

**Keywords:** Machine learning algorithm, Sepsis prediction, Severe sepsis, Diagnostic

## Abstract

**Background:**

Severe sepsis and septic shock are among the leading causes of death in the United States and sepsis remains one of the most expensive conditions to diagnose and treat. Accurate early diagnosis and treatment can reduce the risk of adverse patient outcomes, but the efficacy of traditional rule-based screening methods is limited. The purpose of this study was to develop and validate a machine learning algorithm (MLA) for severe sepsis prediction up to 48 h before onset using a diverse patient dataset.

**Methods:**

Retrospective analysis was performed on datasets composed of de-identified electronic health records collected between 2001 and 2017, including 510,497 inpatient and emergency encounters from 461 health centers collected between 2001 and 2015, and 20,647 inpatient and emergency encounters collected in 2017 from a community hospital. MLA performance was compared to commonly used disease severity scoring systems and was evaluated at 0, 4, 6, 12, 24, and 48 h prior to severe sepsis onset.

**Results:**

270,438 patients were included in analysis. At time of onset, the MLA demonstrated an AUROC of 0.931 (95% CI 0.914, 0.948) and a diagnostic odds ratio (DOR) of 53.105 on a testing dataset, exceeding MEWS (0.725, *P* < .001; DOR 4.358), SOFA (0.716; *P* < .001; DOR 3.720), and SIRS (0.655; *P* < .001; DOR 3.290). For prediction 48 h prior to onset, the MLA achieved an AUROC of 0.827 (95% CI 0.806, 0.848) on a testing dataset. On an external validation dataset, the MLA achieved an AUROC of 0.948 (95% CI 0.942, 0.954) at the time of onset, and 0.752 at 48 h prior to onset.

**Conclusions:**

The MLA accurately predicts severe sepsis onset up to 48 h in advance using only readily available vital signs extracted from the existing patient electronic health records. Relevant implications for clinical practice include improved patient outcomes from early severe sepsis detection and treatment.

## Background

Severe sepsis and septic shock are a dysregulated response to infection, and they are among the leading causes of death in the United States. Epidemiologic estimates have suggested that over 1 million patients are diagnosed with sepsis annually, with case fatality rates exceeding 10% [1, 2]. The cost of treating sepsis is estimated to be $16.7 billion per year, making sepsis one of the most expensive conditions to diagnose and treat [2, 3].

Multiple studies have shown that accurate early diagnosis and treatment, including sepsis bundle compliance, can reduce the risk of adverse patient outcomes from severe sepsis and septic shock [4–6]. Earlier detection and more accurate recognition of patients at high risk of developing severe sepsis or septic shock provide a valuable window for effective sepsis treatments. However, the heterogeneous nature of possible infectious insults and the diversity of host response often make sepsis difficult to recognize in a timely manner [7]. Studies that have attempted to target the risk-factors associated with sepsis onset reveal that sepsis is not a uniform condition. For example, oncology patients are nearly ten times more likely to develop sepsis when compared to patients with no cancer history [8], and patients with sepsis that developed during hospitalization experience a 23% higher mortality rate than patients with community-acquired sepsis [9, 10].

New definitions intended to improve the clinical recognition of sepsis have been proposed [11, 12] because the previous use of screening based on Systemic Inflammatory Response Syndrome (SIRS) criteria was found to be nonspecific [13]. However, SIRS-based sepsis screening is still used in many clinical settings. In addition to SIRS, other rule-based patient decompensation screening tools commonly used for the detection or prediction of sepsis in clinical practice include the Sequential (Sepsis-Related) Organ Failure Assessment (SOFA) score [14] and the Modified Early Warning Score (MEWS) [15]. These methods generate risk scores by manual tabulation of various patient vital signs and laboratory results and have been validated for severe sepsis detection in a variety of studies [16–19]. Efficacy of these scores is limited in part because they do not leverage trends in patient data over time, or correlations between measurements. Some scoring systems, such as SOFA, are not widely applicable outside of the ICU and often require laboratory values that are not rapidly available [20]. While several major EHR systems now have automated sepsis surveillance tools available to their clients [21, 22], these alert tools are rules-based and suffer from low specificity.

Machine learning-based screening methods represent a viable alternative to rules-based screening tools such as MEWS, SIRS, and SOFA, because machine learning algorithms (MLAs) can process complex tasks and large amounts of data. A recent meta-analysis has demonstrated the accuracy of MLAs to predict sepsis and septic shock onset in retrospective studies [23]. However, although a number of machine learning-based algorithms have been developed for sepsis screening [24–29], these models often require extensive training data and laboratory test results [30–32], and some require specialist annotation and the interpretation of clinical notes. These tools have also been limited by a lack of external [24, 26, 31, 33] and real-world [25] validation. Current best practices for reporting and implementing ML-based prediction methods stress the importance of validation on external data, specifically data collected from institutions not used to develop the model [34]. Such validation helps to determine how the model will perform on novel populations and in new clinical settings, and assesses whether the model is overfit to the development dataset. However, while there is a growing expectation that MLAs developed for medical diagnoses are externally validated [35], a meta-analysis of studies using machine-learning-based approaches to predict sepsis reported on only three studies that validated their models on external datasets [36].

In response to the need for externally validated machine learning-based sepsis screening methods, this study evaluates the performance of our MLA which predicts and detects severe sepsis using data extracted from patient Electronic Health Records. It is important that sepsis prediction MLAs have generalizability to different clinical settings and are capable of high performance scores on a diverse dataset, without requiring extensive retraining. For the current study, we assembled a large and diverse retrospective dataset containing inpatient and emergency department patient data from institutions spanning large academic centers to small community hospitals across the continental United States. Performance metrics of the algorithm were evaluated and compared against common rule-based methods using retrospective patient data from 461 hospitals and an external validation data set from Cabell Huntington Hospital. To address the growing need for rigorous external validation on diverse datasets [35], this algorithm was developed and evaluated on significantly larger and more diverse datasets than previously investigated [37–43].

## Methods

### Dataset

The Dascena Analysis Dataset (DAD) and the Cabell Huntington Hospital Dataset (CHHD) were used for retrospective algorithm development, training and testing. The DAD served as the primary development and validation set, and is comprised of 489,850 randomly-selected inpatient and emergency department encounters obtained from de-identified EHR records at 461 total academic and community hospitals across the continental United States. Data contributions to the DAD are imbalanced between hospitals. Data were collected between 2001 and 2015, with the majority of encounters occurring between 2014 and 2015. Details about all hospitals are provided in Additional file 1: Table S1. The CHHD served as an external validation set, and includes 20,647 inpatient and emergency encounters from Cabell Huntington Hospital (Huntington, WV) collected during 2017.

In compliance with the Health Insurance Portability and Accountability Act (HIPAA), all patient information was de-identified prior to retrospective analysis. All data collection was passive and did not have an impact on patient safety.

### Patient measurements and inclusion criteria

All sexes and ethnicities were included in this study. Data was analyzed for only adult EHR records (ages 18 and over) from inpatient (including critical care) wards and emergency department admissions.

**Fig. 1:**
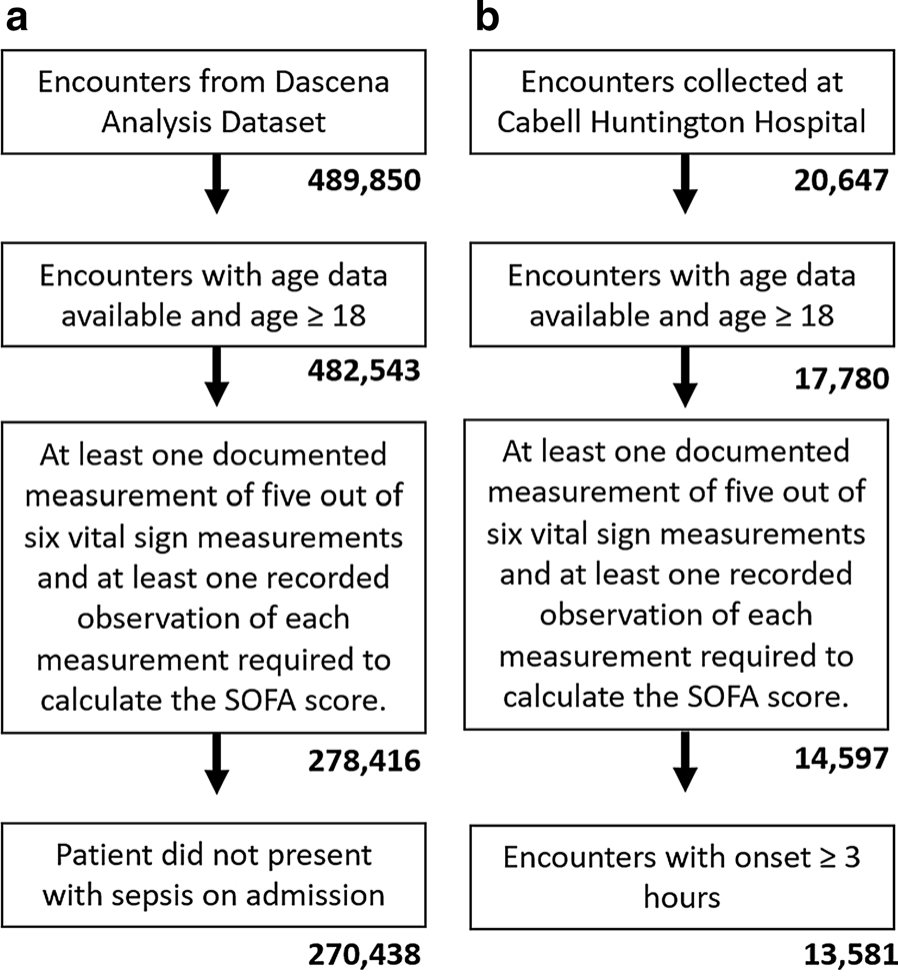
Inclusion criteria for patient encounters. **a** Dascena Analysis Dataset (DAD) and **b** Cabell Huntington Hospital Dataset (CHHD)

For inclusion in the retrospective analysis, patient records were required to contain at least one documented measurement of five out of six vital sign measurements, including: heart rate, respiration rate, temperature, diastolic and systolic blood pressure, and SpO_2_. We also required at least one recorded observation of each measurement required to calculate the SOFA score, including Glasgow Coma Scale, PaO_2_/FiO_2_, bilirubin level, platelet counts, creatinine level, and mean arterial blood pressure or administration of vasopressors (see “Calculating comparators” section below). All patients who presented with sepsis on admission were excluded. These criteria resulted in the inclusion of 270,438 patients from the DAD and 13,581 from the CHHD (Fig. 1 and Additional file 1: Table S2). Patients were divided into subgroups based on hospital length of stay in order to assess MLA performance at several predetermined prediction times (4, 6, 12, 24, and 48 h before onset). Patients were included in analysis only if their length of stay exceeded the tested prediction time. This resulted in decreasing subgroup size as prediction time was increased. For each prediction time, patients who became severely septic within 2 h of the prediction window were excluded. This ensured the presence of adequate data with which to train and test the algorithm for each prediction task. To ensure that these exclusion criteria did not introduce selection bias into the study population, we compared demographic and clinical measurements among included and excluded patients to compare the distribution of patient characteristics and illness severity. For any patient with a stay exceeding 2000 h, the last 2000 h of hospital data were used for the studyin order to limit the size of data analysis matrices and control for atypical patient encounters. See Additional file 1: Fig. S2 for additional details of patient data processing.

### Binning and imputation

For retrospective analysis, MLA predictions were made using only patient age, systolic blood pressure, diastolic blood pressure, heart rate, temperature, respiratory rate and SpO_2_ measurements. These measurements were chosen because these vital signs are commonly available and frequently sampled. The average number of readings per hour for septic and non-septic patients in each dataset are presented in Additional file 1: Table S3. These measurements were binned by the hour for each included patient, beginning at the time of the patient’s first recorded measurement and ending with the last whole hour of available data observed before the patient’s final measurement. Measurements were each binned into 1-h intervals and averaged to provide a single hourly value, which minimizes information fed to the classifier regarding sampling frequency. Binning the data into intervals generates a discrete time series with consistent time steps, which are more readily processed by the algorithm. Missing values were filled using last-one carry forward (LOCF) imputation, wherein the most recent observation of that measurement is used to replace the missing value. This imputation method is appropriate for clinical measurements, because observations of a given vital sign are expected to be highly dependent on previous observations [44–46].

### Gold standard

For retrospective analysis, we defined our severe sepsis gold standard by the presence of International Classification of Diseases, Ninth Revision (ICD-9) code 995.9x. Identifying sepsis through the explicit use of ICD codes alone is known to have high specificity with low sensitivity [47]; for the purposes of this study we prioritized specificity to ensure that all patients labeled as septic truly experienced sepsis. To determine the onset time of severe sepsis, we identified the first time at which “organ dysfunction caused by sepsis,” with sepsis defined as “the presence of two or more SIRS criteria paired with a suspicion of infection” [3] was present in the patient chart. This is similar to the Sepsis-3 definition of sepsis as “a life-threatening organ dysfunction caused by a dysregulated host response to infection,” where organ dysfunction is defined as “an acute change in total SOFA score ≥ 2 points consequent to the infection [12].” We defined the onset time as the first time at which two SIRS criteria and at least one organ dysfunction criteria (Additional file 1: Table S4) were met within the same hour. For patients who never developed sepsis, onset time was selected at random from the patient stay. For patients who never developed sepsis, onset time was selected at random from the patient stay.

### Calculating comparators

In this retrospective analysis, we fixed severe sepsis identification score thresholds of 2, 2, and 1 for MEWS, SOFA, and SIRS criteria, respectively. In other words, a MEWS score ≥ 2 indicates a patient would be categorized by MEWS as septic. These thresholds were selected to produce a sensitivity closest to 0.80. A constant sensitivity close to 0.80 was chosen for all systems to facilitate comparison; the threshold for sepsis identification using SIRS is therefore different from the SIRS threshold used in the gold standard onset time definition above. Similarly, to facilitate comparison of the MLA with other methods, we selected a fixed point on the Receiver Operating Characteristic (ROC) curve of the MLA with sensitivity near 0.80. This enabled table-based comparisons of specificity while holding sensitivity relatively constant. All comparators were calculated for severe sepsis detection at the time of onset assigned by the gold standard using the DAD test dataset. We compared the performance of the MLA and rules-based systems using the area under the ROC (AUROC) curve. The following additional performance metrics were also calculated for the MLA and comparators: accuracy, diagnostic odds ratio (DOR) and positive and negative likelihood ratios (LR+ and LR−).

### The machine learning algorithm

We constructed our classifier using gradient boosted trees, implemented in Python (Python Software Foundation, https://www.python.org/) with the XGBoost package [48]. Predictions were generated from patient age and the binned values for the vital signs of systolic blood pressure, diastolic blood pressure, heart rate, temperature, respiratory rate and SpO_2_ at prediction time, 1 h before prediction time and 2 h before prediction time. Where appropriate, we also concatenated the differences in measurement values between those time steps. In the data matrices, each clinical feature thus represented between 3 and 5 columns. Values were concatenated into a feature vector with fifteen elements. All data processing was performed using Python software [49]. An ensemble of decision trees was constructed using the gradient boosted trees approach, after which the ensemble made a prediction based on an aggregate of these scores. In this way, at prediction time, the gradient boosted tree ensemble was able to access trend information and covariance structure with respect to time window. This procedure of transforming time series problems into supervised learning problems has also been detailed in our previous work [46]. XGBoost controlled for expected class imbalance in the data. Minority class scaling was employed within the algorithm, where instances of the minority class were given weight inversely-proportional to their prevalence, which effectively trained the models on approximately balanced data. Tree branching was determined evaluating the impurity improvements gained from potential partitions, and patient risk scores were determined by their final categorization in each tree. We limited tree branching to six levels, included no more than 1000 trees in the final ensemble, and set the XGBoost learning rate to 0.1. These hyperparameters were chosen to align with previous work and justified in the context of the present data with a coarse grid search using training data [38].

### Study design

For retrospective analysis, model performance was evaluated by using ten-fold cross validation procedures for training, an independent, hold-out test set for evaluation, and an external validation set. These three levels of validation allowed us to examine the performance of the trained models in different data distribution settings; the distribution of the validation data varied from very similar to the training data, in the case of tenfold cross validation, to very different, in the case of external validation. To generate the independent, hold-out test set, we randomly selected 80% of the DAD to be used for training, while reserving the remaining 20% of the dataset as the independent, hold-out test set. On the training data, we performed tenfold cross validation by then further dividing the training set into tenths, training the algorithm on nine of these tenths and assessing its performance on the remaining tenth. We repeated this process ten times, using each possible combination of training and testing folds within the training dataset. We then assessed each of the resulting ten models on the independent, hold-out test set. Reported performance metrics for the hold out test set are the average performance of each of these ten models on the hold-out test set. The reported score thresholds, the MLA score at which a patient was deemed to be positive for severe sepsis, is an average of the threshold score in each of the ten models generated in the tenfold cross validation training procedures. These score thresholds were determined using the fixed operating point on the ROC curve, near sensitivity of 0.80. The CHHD served as the external validation set, which was used to further assess each of the ten models and examine the generalizability of the approach to different patient demographics and data collection methods.

### Statistical analysis

MLA AUROC values were compared to those of SIRS, SOFA and MEWS using two-sample *t* tests at 95% confidence. *P*-values for algorithm comparisons with all comparator systems were found to be statistically significant at *P* < 0.001.

## Results

Patient demographic data from the DAD, which consists of inpatient and emergency department encounters from 461 academic and community US hospitals, and the CHHD external validation dataset are presented in Table 1. The overall prevalence of severely septic patients in this population was 4.3%. Among those patients classified as septic, the mean age was 62 years (49.5% male vs 44.7% female). A comparison of demographic and clinical characteristics among included and excluded patients demonstrates that the included sample is representative of the entire patient population (Additional file 1: Table S5).

**Table 1:** Demographics table

	DAD		CHHD	
	Septic	Non-septic	Septic	Non-septic
Total number	20,876	468,974	182	20,465
Age (SD)	62.4 (17.0)	55.62 (18.7)	50.5 (24.2)	40.4 (23.0)
Male	10,326 (49.5%)	221,029 (47.1%)	69 (37.9%)	7470 (36.5%)
Female	9325 (44.7%)	219,866 (46.9%)	88 (48.4%)	10,595 (51.8%)
Sex Unknown	1225 (5.9%)	28,079 (6.0%)	25 (13.7%)	2400 (11.7%)
White	9394 (45.0%)	145,891 (31.1%)	100 (54.9%)	11,854 (57.9%)
Black	1150 (5.5%)	20,158 (4.3%)	9 (4.9%)	764 (3.7%)
Hispanic	1090 (5.2%)	33,944 (7.2%)	0 (0.0%)	2 (0.0%)
Asian American	250 (1.2%)	3020 (0.6%)	1 (0.5%)	18 (0.1%)
Race/Ethnicity Unknown	8992 (43.1%)	265,961 (56.7%)	72 (39.6%)	7821 (38.2%)
Temperature	36.9 (0.7)	36.8 (0.5)	36.9 (0.3)	36.8 (0.2)
Respiratory rate	21.1 (4.6)	18.7 (4.1)	20.8 (7.5)	18.1 (5.1)
Systolic blood pressure	115.2 (17.7)	123.9 (17.1)	119.1 (16.7)	125.5 (16.7)
Diastolic blood pressure	61.2 (11.8)	68.6 (11.6)	66.6 (9.6)	73.2 (10.5)
Heart rate	90.9 (14.9)	83.8 (17.0)	93.8 (16.7)	85.4 (17.1)
Lactate	1.6 (1.6)	1.43 (1.1)	2.6 (2.0)	1.9 (1.6)
Creatinine	1.6 (1.4)	1.2 (1.2)	1.7 (1.8)	1.5 (2.6)
International normalized ratio (INR)	1.2 (0.9)	1.0 (0.7)	1.4 (0.8)	1.1 (0.4)
Platelets	204.0 (113.0)	220.4 (95.4)	238.5 (105.1)	239.6 (75.0)
SpO_2_	96.4 (3.1)	97.0 (2.3)	96.9 (1.6)	97.64 (1.3)
White blood count	12.8 (5.5)	10.5 (4.2)	8.6 (1.4)	8.2 (1.7)
PaO_2_	115.0 (36.6)	131.2 (62.0)	95.6 (27.8)	102.6 (45.3)
Bilirubin	1.1 (1.4)	0.8 (0.9)	1.3 (2.46)	0.7 (1.2)
FiO_2_	49.7 (23.8)	46.8 (23.6)	47.4 (20.4)	42.0 (18.1)
pH	7.4 (0.1)	7.4 (0.1)	7.4 (0.1)	7.4 (0.1)

Demographic and clinical characteristics of patients included in the Dascena analysis dataset (DAD) and CHH dataset (CHHD)

The detailed numerical results in Table 2 show that the MLA provided a superior severe sepsis predictor compared with alternative scoring systems of MEWS, SOFA, and SIRS. AUROC represents the area under the ROC curves, which plot sensitivity (the fraction of severe sepsis patients that were classified as severe sepsis) as a function of 1 − specificity (the fraction of severe sepsis-negative patients that were classified as severe sepsis).

**Table 2:** Comparison table of performance metrics for MLA to standard scoring systems, at time of severe sepsis onset

	MLA ≥ 0.029 DAD training	MLA ≥ 0.030 DAD testing	MLA ≥ 0.017 CHH external validation	MEWS ≥ 2 DAD testing	SOFA ≥ 2 DAD testing	SIRS ≥ 1 DAD testing
AUROC (SD)	0.931 (0.01)	0.930 (0.01)	0.948 (0.01)	0.725	0.716	0.655
*P* value (MLA vs comparator)	–	–	–	*P* < 0.001	*P* < 0.001	*P* < 0.001
Sensitivity	0.800	0.800	0.800	0.845	0.750	0.868
Specificity	0.926	0.933	0.921	0.444	0.554	0.334
Accuracy	0.923	0.929	0.920	0.608	0.645	0.646
DOR	53.105	56.508	47.532	4.358	3.720	3.290
LR+	11.411	12.110	10.306	1.521	1.680	1.303
LR−	0.216	0.215	0.217	0.349	0.452	0.396

Detailed performance metrics for the Machine Learning Algorithm (MLA) and rules-based systems taken at the time of severe sepsis onset, using the Dascena Analysis Dataset for training and testing and the Cabell Huntington Hospital dataset for external validation. The score threshold reported for the MLA is the average over rounds of ten-fold cross-validation. AUROC for MLA versus comparators was performed using two-sample t-tests at 95% confidence. *AUROC* area under the receiver operating characteristic, *MEWS* Modified Early Warning Score, *SOFA* Sequential Organ Failure Assessment, *SIRS* Systemic Inflammatory Response Syndrome, *DOR* diagnostic odds ratio, *LR* likelihood ratio

At 95% confidence, the MLA demonstrated a higher severe sepsis detection AUROC (0.931, 0.930, 0.948 on training, testing, and external validation validation datasets respectively) than MEWS (0.725; *P* < 0.001), SOFA (0.716; *P* < 0.001), and SIRS (0.655; *P* < 0.001) (Table 2). Detailed performance metrics for all scoring systems at time of severe sepsis onset are presented in Table 2. Accuracy is a standard performance metric for binary classification and represents the proportion of correct classifications out of all classifications made. DOR represents the odds of a severe sepsis prediction for severe sepsis patients relative to patients who do not have severe sepsis. Likelihood ratios are also included as indicators of diagnostic accuracy. Here, LR+ represents the ratio of the probability that a severe sepsis-positive classification will be assigned to severe sepsis patients to the probability that a severe sepsis-positive classification will be assigned to patients who do not have severe sepsis. LR− represents the ratio of the probability that a severe sepsis-negative classification will be assigned to severe sepsis patients to the probability that a severe sepsis-negative classification will be assigned to patients who do not have severe sepsis. In addition to AUROC values, the MLA maintained superior performance metric scores (vs all comparators) for Specificity, Accuracy, DOR, LR+, and LR− (Table 2).

**Fig. 2:**
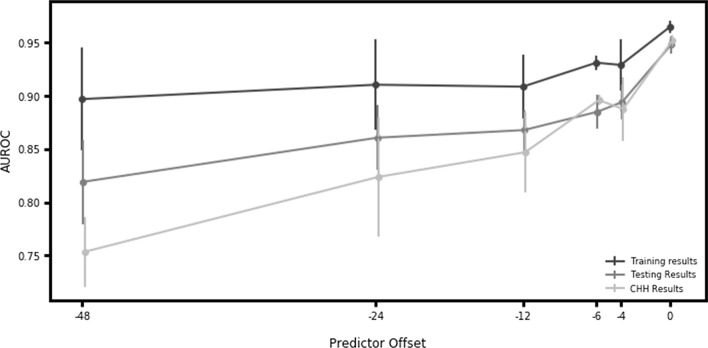
AUROC over time. Depicts performance of the MLA in predicting the onset of severe sepsis at 0, 4, 6, 12, 24 and 48 h before severe sepsis onset. “Training Set” results were derived from the DAD, “Testing Set” results were derived from the hold out data from the DAD, and the “External Validation Set” was derived from the independent CHHD

Additionally, the MLA maintained a superior AUROC for all prediction windows as compared to all onset-time rules-based scoring systems; at 48 h prior to severe sepsis onset, the MLA demonstrated an AUROC value of 0.75 on the external validation dataset (Fig. 2). Detailed performance metrics for the MLA at all prediction windows are presented in Additional file 1: Tables S6, S7, and S8 for the training set, testing set, and external validation set, respectively.

We ranked the feature importance for severe sepsis detection and prediction using the MLA using average entropy gain for each feature. Feature importance varied significantly by prediction window (Additional file 1: Fig. S1).

The standard deviation for the external validation dataset**,** which quantifies variability in patient populations [50], became larger at longer look-ahead times. This indicates increased variation in the performance of the algorithm at longer look-ahead times in the external validation set.

## Discussion

The machine learning algorithm more accurately detected the onset of severe sepsis developed during hospitalization than the frequently used rules-based patient decompensation screening tools MEWS, SOFA, and SIRS. While used for sepsis screening in many clinical settings, these tools are not designed to exploit information from trends in patient data, and demonstrate suboptimal specificity [14–20]. Up to 48 h before onset, the MLA demonstrated higher AUROC and specificity than the commonly used rules-based sepsis screening systems when evaluated at the time of sepsis onset. The algorithm used only patient age and six vital signs extracted from the patient EHR, and did not require manual data entry or calculation. The accuracy of the MLA for early severe sepsis prediction, together with the minimal patient data required, suggests that this system may improve severe sepsis detection and patient outcomes in prospective clinical settings over the use of a rules-based system. The high specificity of the MLA may also reduce alarm fatigue, a known patient safety hazard [51].

Recent studies using MLAs to provide early detection and prediction of sepsis, severe sepsis and/or septic shock include Long Short-Term Memory (LSTM) neural network based algorithms [31, 33], the recurrent neural survival model “DeepAISE” [32], and the random-forest classifier “EWS 2.0” [30]. In their clinical practice impact study, Giannini et al. [30] developed and implemented the EWS 2.0 model to predict severe sepsis and septic shock. Although the alerting system was able to make a modest impact on clinical practices, the reported sensitivity was 26% and the average prediction time prior to onset was approximately 6 h [30]. Among recent studies focusing on longer horizon predictions, Fagerström et al.’s [33] LSTM model, “LiSep LSTM”, predicted septic shock with an AUROC of 0.83 up to 40 h prior to onset, and the model developed by Lauritsen et al. [31] used a deep learning approach to predict sepsis onset 24 h prior to onset with an AUROC of 0.76. Although the MLAs used in these studies were not validated on an external dataset, limiting generalizability of the models, they illustrate the utility of neural network-based algorithms towards long horizon sepsis predictions. In a recently posted preprint by Shashikumar et al. [32], an externally validated recurrent neural survival model, DeepAISE, achieved high performance metrics for prediction sepsis up to 12 h prior to onset. While the DeepAISE model generated predictions using a large number of features, the MLA in our study was designed to provide accurate long-horizon predictions that require only minimal inputs.

In this study, the algorithm was tested on a large and diverse retrospective dataset containing inpatient and emergency department patient data from 461 teaching and non-teaching hospitals in the US. This dataset includes patient data from intensive care unit and floor wards, representing a variety of data collection frequencies and care provision levels. This dataset is significantly larger and more diverse than datasets used to develop previous versions of the algorithm, which has been applied to sepsis and severe sepsis detection using only vital sign data in the emergency department, general ward and ICU [37, 40–42] and has been evaluated for its effect on clinical outcomes in a single-center study [39] as well as a randomised clinical trial [38].

Sepsis manifestation can vary depending on factors such as patient race, age, and comorbidities [52]. This is evidenced by a recent study which found that feature selection that accounted for sepsis subpopulations resulted in increased performance of classification models [53], as well as by our prior work predicting severe sepsis in the pediatric subpopulation, which showed that a sepsis prediction algorithm could be successfully tailored to this specific subpopulation [54]. It is therefore important that sepsis detection methods geared towards the general patient population be validated across a diverse population in order to ensure accurate discrimination for all patients. The high performance of the MLA on the diverse dataset utilized in this study indicates that the algorithm may be able to improve patient outcomes in a variety of clinical settings. In addition to strong performance on a hold-out test set, consistent performance on an external validation set demonstrates generalizability to different clinical settings.

While the retrospective analysis incorporated data from a large number of institutions (nearly 10% of US hospitals), we cannot claim generalizability to additional specific settings or populations on the basis of this study. While data in the DAD were collected between 2001 and 2015, the majority of encounters occured between 2014 and 2015. The use of data generated primarily during the years 2014–2015 may limit the generalizability of these results. Generalizability of the retrospective results is also limited by our inclusion criteria requiring that all patients manifesting severe sepsis within 2 h of each prediction window be excluded from the analysis. Because we do not perform any subgroup analyses in the present study, we also cannot verify the generalizability of these results to specific patient subpopulations. Future work investigating performance on subpopulations defined by medical or demographic characteristics is therefore warranted. The required presence of an ICD-9 code to classify a patient as severely septic in our retrospective analysis potentially limits our ability to accurately capture all septic patients in the dataset [47], as any undiagnosed or inaccurately coded patients may have been improperly labeled as non-septic. However, past research has shown ICD-9 coding to be a reasonable means of retrospectively detecting patients with severe sepsis [55, 56]. Further, our gold standard criteria may also limit the accuracy of our severe sepsis onset time analysis, as the time a condition was recorded in the patient chart may not represent the time the condition actually manifested. Finally, because our study is a retrospective analysis of encounters which do not involve the intervention of predictions from the MLA, we must await real-time, prospective evaluation of the algorithm before making claims of impact on clinical practice and patient outcomes.

In this retrospective analysis, we treated severe sepsis detection and prediction as a classification task. While a time-to-event modeling approach would have also been possible, classification methods are significantly more common in the literature [24, 57–60]. By using the same modelling approach, the present study can be readily compared with existing work on sepsis detection models using standard metrics such as AUROC and specificity.

## Conclusion

This study validates a machine learning algorithm for severe sepsis detection and prediction developed with a diverse retrospective dataset containing patient data from 461 academic centers and community hospitals across the US. The algorithm, validated on an external dataset, is capable of predicting severe sepsis onset up to 48 h in advance of onset using only patient age and six frequently collected patient measurements, and demonstrates higher AUROC values and specificity than commonly used sepsis detection methods such as MEWS, SOFA and SIRS, applied at onset.

The accuracy of the sepsis prediction MLA validated in this study, paired with the minimal patient data required for predictions, supports the premise that MLAs can be used to improve severe sepsis detection and patient outcomes in a diversity of medical care facilities and wards, without requiring additional data analyses from clinicians. The high specificity of the MLA in this study may help to reduce alarm fatigue. Relevant potential implications for clinical practice include improved patient outcomes arising from early severe sepsis detection and treatment.

## Supplementary information

**Additional file 1.** Validation of a machine learning algorithm for early severe sepsis prediction: a retrospective study predicting severe sepsis up to 48 h in advance using a diverse dataset from 461 US hospitals.

## Data Availability

Restrictions apply to the availability of the patient data, which were used under license for the current study, and so are not publicly available. The MLA code developed in this study is proprietary and not publicly available.

## Abbreviations

AUROC: Area under the receiver operating characteristic
CHHD: Cabell Huntington Hospital dataset
DAD: Dascena Analysis Dataset
DOR: Diagnostic odds ratio
ICD: International Classification of Diseases
LOCF: Last-one carry forward
LR: Likelihood ratio
MEWS: Modified Early Warning Score
MLA: Machine learning algorithm
SIRS: Systemic Inflammatory Response Syndrome
SOFA: Sequential Organ Failure Assessment
