# Supplementary material for: Validation of a machine learning algorithm for early severe sepsis prediction: a retrospective study predicting severe sepsis up to 48 h in advance using a diverse dataset from 461 US hospitals
Source: BMC Med Inform Decis Mak. 2020 Oct 27;20:276. doi: 10.1186/s12911-020-01284-x (PMC7590695; doi:10.1186/s12911-020-01284-x)
Supplement: Supplementary file 1 — Additional file 1. Validation of a machine learning algorithm for early severe sepsis prediction: a retrospective study predicting severe sepsis up to 48 h in advance using a diverse dataset from 461 US hospitals. [file 12911_2020_1284_MOESM1_ESM.docx]

**SUPPLEMENTAL MATERIALS**

| 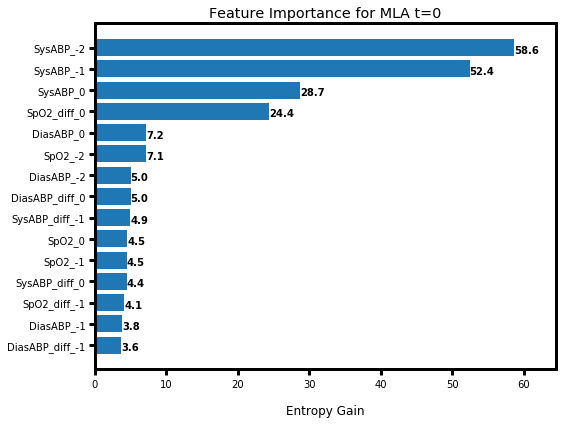A | 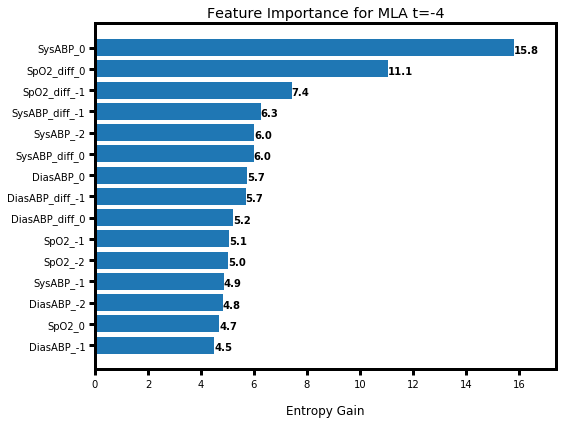B |
| --- | --- |
| 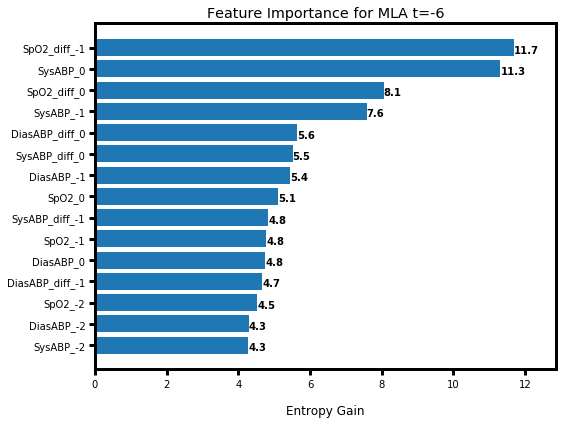C | 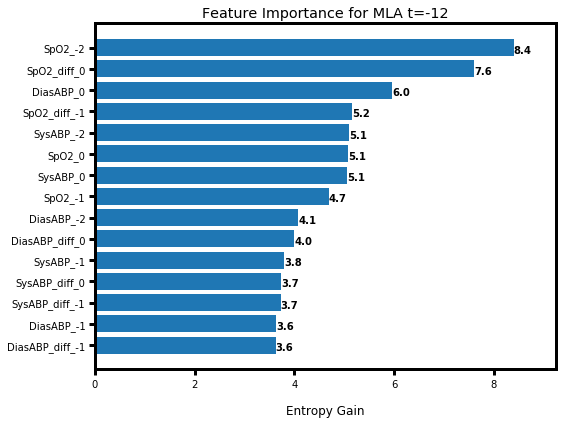D |
| 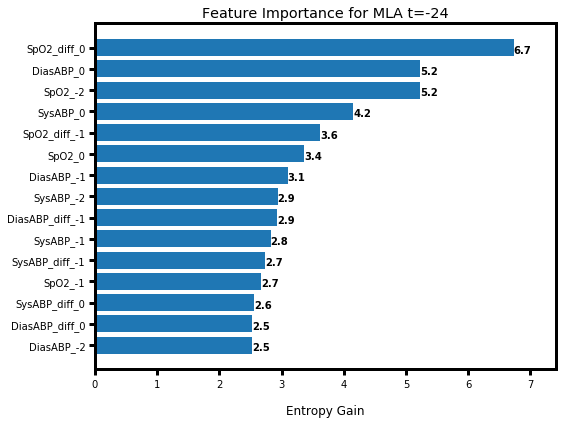E | 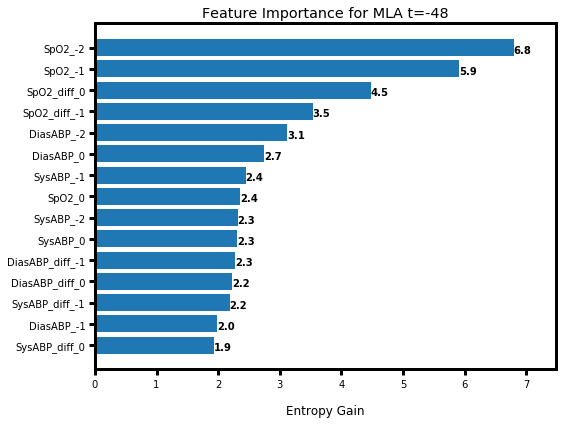F |

**Supplementary Figure 1**: **Average entropy gain for each feature used to generate machine learning sepsis prediction scores** at a) time of onset, b) 4 hours before onset, c) 6 hours before onset, d) 12 hours before onset, e) 24 hours before onset, and d) 48 hours before onset. MLA, machine-learning algorithm; SysABP, systolic ambulatory blood pressure; SpO2, peripheral capillary oxygen saturation; DiasABP, diastolic ambulatory blood pressure; diff, indicates features constructed by taking the difference between a measurement at a given time and the previous hour.

**
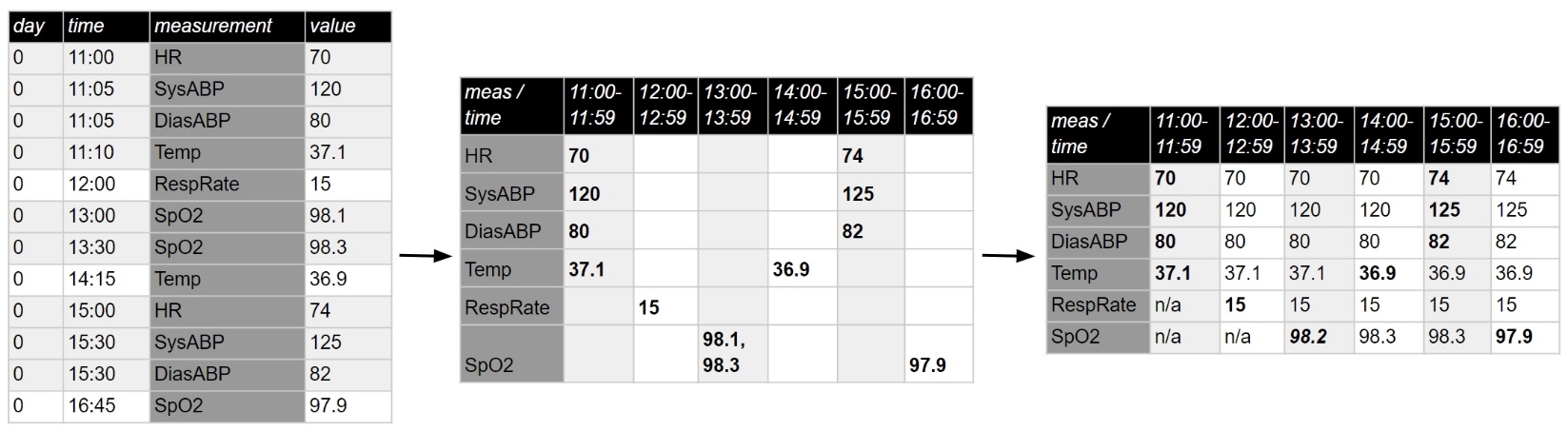
**

**Supplementary Figure 2:** Example of patient data processing showing a) a series of patient measurements over time b) patient measurements after binning and c) patient data as passed to the machine learning algorithm. Abbreviations used: HR: Heart rate. ABP: Arterial blood pressure. SpO_2_: Peripheral oxygen saturation. Temp: Temperature. Measurements are provided in the following units: HR: Beats/minute. SysABP/DiasABP: mmHg. SpO_2_: Percent. Resp rate: respirations/minute. Temp: degrees Celsius.

**Supplementary Table 1:** **Hospital Characteristics**

| **Hospital Characteristic** | **Retrospective Analysis** |
| --- | --- |
| **Geographic Region** |  |
| Northeast | 22 |
| South | 89 |
| Midwest | 108 |
| West | 66 |
| Unknown | 176 |
| **Teaching Status** |  |
| Teaching | 21 |
| Non-teaching | 270 |
| Unknown | 170 |
| **Hospital Size** |  |
| Small (<100 beds) | 87 |
| Medium (100-250 beds) | 86 |
| Large (>250 beds) | 68 |
| Unknown | 220 |

**Supplementary Table 2: Retrospective Dataset Patient Characteristics and Patient Inclusion Criteria**

| **Stages** | **Total Number of Patients** | **Severe Sepsis Patients** |
| --- | --- | --- |
| Unfiltered | 489,850 |  |
| Has age information and age > 18 | 482,543 |  |
| Data for at least one measurement of each of the 5 vitals available | 278,416 |  |
|  |  |  |
| For t = 0 prediction | 270,438 | 9,254 |
| For t = 4 prediction | 266,889 | 5,705 |
| For t = 6 prediction | 266,079 | 4,895 |
| For t = 12 prediction | 264,525 | 3,341 |
| For t = 24 prediction | 263,323 | 2,139 |
| For t = 48 prediction | 262,494 | 1,310 |

**Supplementary Table 3: Average number of feature recordings per hour for septic and non-septic patients in the DAD and CHH datasets.**

|  | **DAD** | | **CHH** | |
| --- | --- | --- | --- | --- |
|  | **Septic** | **Non-Septic** | **Septic** | **Non-Septic** |
| **DiasABP** | 1.803 | 1.106 | 0.385 | 0.402 |
| **SysABP** | 1.803 | 1.106 | 0.391 | 0.402 |
| **HR** | 4.419 | 5.740 | 0.588 | 0.452 |
| **Temp** | 0.776 | 0.540 | 0.155 | 0.250 |
| **RespRate** | 4.045 | 5.044 | 0.433 | 0.380 |
| **SpO2** | 4.216 | 5.434 | 0.419 | 0.389 |
| **Lactate** | 0.479 | 1.048 | 0.336 | 0.354 |
| **Creatinine** | 0.110 | 0.875 | 0.044 | 0.085 |
| **INR** | 0.090 | 0.169 | 0.072 | 0.040 |
| **Platelets** | 0.094 | 0.091 | 0.039 | 0.022 |
| **WBC** | 0.094 | 0.091 | 0.026 | 0.048 |
| **PaO2** | 0.292 | 0.830 | 0.098 | 0.146 |
| **Bilirubin** | 0.124 | 0.147 | 0.046 | 0.021 |
| **FiO2** | 0.824 | 1.302 | 0.947 | 0.733 |
| **pH** | 0.296 | 0.720 | 0.098 | 0.147 |

**Supplementary Table 4:** **SIRS Criteria and organ dysfunction criteria** used to identify positive cases of severe sepsis.

| **SIRS Criteria** | **Organ Dysfunction Criteria** |
| --- | --- |
| - Heart rate > 90 beats/ min, - Body temperature > 38 ℃ or < 36 ℃, - Respiratory rate >20 breaths/min or PaCO_2_ < 32 mmHg, and - White blood cell count > 12,000 cells/μL or < 4,000 cells/μL. | - Lactate > 2 mmol/L - Systolic blood pressure < 90 mmHg - Urine output < 0.5 mL/kg, over two hours, prior to organ dysfunction after fluid resuscitation - Creatinine > 2 mg/dL without renal insufficiency or chronic dialysis - Bilirubin > 2 mg/dL without having liver disease or cirrhosis - Platelet count < 100,000 μL - International normalized ratio > 1.5 - PaO2/FiO2 < 200 in addition to pneumonia < 250 with acute kidney injury but without pneumonia |

**Supplementary Table 5: Distribution of demographic and clinical characteristics of DAD patients included and excluded from the study sample.**

|  | **DAD Patient Encounters** | |
| --- | --- | --- |
|  | **Included** | **Excluded** |
| **Total Number** | 245,257 | 244,593 |
| **Age (SD)** | 54.22 (18.64) | 57.65 (18.61) |
| **Male (%)** | 107,114 (43.7%) | 124,241 (50.8%) |
| **Female (%)** | 110,323 (45.0%) | 118,868 (48.6%) |
| **Unknown (%)** | 27820 (11.3%) | 1484 (0.6%) |
| **White (%)** | 32,628 (13.3%) | 122,657 (50.1%) |
| **Black (%)** | 4120 (1.7%) | 17188 (7.0%) |
| **Hispanic (%)** | 15074 (6.1%) | 19960 (8.2%) |
| **Asian American (%)** | 733 (0.3%) | 2537 (1.0%) |
| **Unknown (%)** | 192702 (78.6%) | 82251 (33.6%) |
| **Temp (SD)** | 36.74 (0.85) | 36.80 (3.22) |
| **RespRate (SD)** | 18.20 (24.03) | 20.19 (10.17) |
| **SysABP (SD)** | 123.88 (18.48) | 122.91 (20.89) |
| **DiasABP (SD)** | 68.70 (14.97) | 69.69 (14.59) |
| **HR (SD)** | 81.93 (70.77) | 86.33 (35.52) |
| **Lactate (SD)** | 1.36 (0.99) | 1.48 (1.25) |
| **Creatinine (SD)** | 0.95 (0.92) | 1.17 (1.08) |
| **INR (SD)** | 0.95 (0.70) | 1.05 (0.89) |
| **Platelets (SD)** | 214.50 (95.97) | 218.37 (98.41) |
| **SpO2 (SD)** | 97.03 (2.27) | 96.41 (3.35) |
| **WBC (SD)** | 11.42 (4.39) | 10.48 (4.46) |
| **PaO2 (SD)** | 1.72 (10.80) | 2.02 (12.20) |
| **Bilirubin (SD)** | 1.06 (2.29) | 0.99 (2.12) |
| **FiO2 (SD)** | 46.03 (22.28) | 41.90 (23.37) |
| **pH (SD)** | 7.37 (0.11) | 7.38 (0.10) |

**Supplementary Table 6: Training MLA Performance Metrics Table, at measured prediction windows.** Detailed performance metrics for the machine learning algorithm (MLA) at 4, 6, 12, 24, and 48 hours before severe sepsis onset on the DAD training dataset. The reported score thresholds are averages over the corresponding rounds of ten-fold cross-validation. DOR: Diagnostic Odds Ratio; LR: Likelihood Ratio.

|  | **MLA  ≥ 0.029 (t = -0 hrs)** | **MLA  ≥ 0.026 (t = -4 hrs)** | **MLA  ≥ 0.042 (t = -6 hrs)** | **MLA  ≥ 0.051 (t = -12 hrs)** | **MLA   ≥ 0.032 (t = -24 hrs)** | **MLA ≥ 0.072 (t = -48 hrs)** |
| --- | --- | --- | --- | --- | --- | --- |
| **AUROC (SD)** | 0.931 (0.01) | 0.885 (0.02) | 0.875 (0.01) | 0.861 (0.03) | 0.846 (0.04) | 0.840 (0.05) |
| **Sensitivity** | 0.800 | 0.800 | 0.800 | 0.801 | 0.799 | 0.794 |
| **Specificity** | 0.926 | 0.799 | 0.782 | 0.752 | 0.701 | 0.703 |
| **Accuracy** | 0.923 | 0.799 | 0.782 | 0.755 | 0.702 | 0.717 |
| **DOR** | 53.105 | 16.49 | 14.91 | 12.888 | 9.617 | 9.780 |
| **LR+** | 11.411 | 4.095 | 3.784 | 3.376 | 2.729 | 2.784 |
| **LR-** | 0.216 | 0.251 | 0.257 | 0.266 | 0.288 | 0.296 |

**Supplementary Table 7: Testing MLA Performance Metrics Table, at measured prediction windows.** Detailed performance metrics for the machine learning algorithm (MLA) at 4, 6, 12, 24, and 48 hours before severe sepsis onset on the DAD testing dataset. The reported score thresholds are averages over the corresponding rounds of ten-fold cross-validation. DOR: Diagnostic Odds Ratio; LR: Likelihood Ratio.

|  | **MLA  ≥ 0.030 (t = -0 hrs)** | **MLA  ≥ 0.027 (t = -4 hrs)** | **MLA  ≥ 0.042 (t = -6 hrs)** | **MLA  ≥ 0.051 (t = -12 hrs)** | **MLA   ≥ 0.033 (t = -24 hrs)** | **MLA ≥ 0.071 (t = -48 hrs)** |
| --- | --- | --- | --- | --- | --- | --- |
| **AUROC (SD)** | 0.930 (0.01) | 0.883 (0.02) | 0.882 (0.02) | 0.869 (0.02) | 0.857 (0.03) | 0.827 (0.04) |
| **Sensitivity** | 0.800 | 0.800 | 0.800 | 0.800 | 0.800 | 0.797 |
| **Specificity** | 0.933 | 0.806 | 0.791 | 0.759 | 0.740 | 0.672 |
| **Accuracy** | 0.929 | 0.806 | 0.791 | 0.761 | 0.745 | 0.678 |
| **DOR** | 56.508 | 16.851 | 15.347 | 12.984 | 11.758 | 8.102 |
| **LR+** | 12.110 | 4.164 | 3.869 | 3.397 | 3.147 | 2.439 |
| **LR-** | 0.215 | 0.248 | 0.253 | 0.264 | 0.270 | 0.303 |

**Supplementary Table 8: External Validation MLA Performance Metrics Table, at measured prediction windows.** Detailed performance metrics for the machine learning algorithm (MLA) at 4, 6, 12, 24, and 48 hours before severe sepsis onset on the CHHD external validation dataset. The reported score thresholds are averages over the corresponding rounds of ten-fold cross-validation. DOR: Diagnostic Odds Ratio; LR: Likelihood Ratio.

|  | **MLA  ≥ 0.017 (t = -0 hrs)** | **MLA  ≥ 0.015 (t = -4 hrs)** | **MLA  ≥ 0.033 (t = -6 hrs)** | **MLA  ≥ 0.045 (t = -12 hrs)** | **MLA   ≥ 0.028 (t = -24 hrs)** | **MLA ≥ 0.069 (t = -48 hrs)** |
| --- | --- | --- | --- | --- | --- | --- |
| **AUROC (SD)** | 0.948 (0.01) | 0.880 (0.03) | 0.861 (<0.01) | 0.862 (0.04) | 0.811 (0.06) | 0.752 (0.03) |
| **Sensitivity** | 0.800 | 0.800 | 0.798 | 0.809 | 0.799 | 0.733 |
| **Specificity** | 0.921 | 0.704 | 0.673 | 0.671 | 0.539 | 0.503 |
| **Accuracy** | 0.920 | 0.706 | 0.681 | 0.694 | 0.559 | 0.525 |
| **DOR** | 47.532 | 9.640 | 8.246 | 8.783 | 6.192 | 3.815 |
| **LR+** | 10.306 | 2.728 | 2.464 | 2.467 | 2.020 | 1.607 |
| **LR-** | 0.217 | 0.284 | 0.301 | 0.284 | 0.441 | 0.569 |
